# How health care professionals confront and solve ethical dilemmas – a tale of two countries: Slovenia and Croatia

**DOI:** 10.3325/cmj.2021.62.120

**Published:** 2021-04

**Authors:** Štefan Grosek, Rok Kučan, Jon Grošelj, Miha Oražem, Urh Grošelj, Vanja Erčulj, Jaro Lajovic, Blaž Ivanc, Milivoje Novak, Larisa Prpić Massari, Suzana Mimica Matanović, Vesna Čerfalvi, Julije Meštrović, Ana Borovečki

**Affiliations:** 1 Neonatology Section, Department of Perinatology, Division of Obstetrics and Gynecology, University Medical Centre Ljubljana, Slovenia; 2 Department of Pediatric Intensive Therapy, Division of Surgery, University Medical Centre Ljubljana, Slovenia; 3 Department of Pediatrics, Faculty of Medicine, University of Ljubljana, Ljubljana, Slovenia; 4 University Children’s Hospital, University Medical Centre Ljubljana, Slovenia; 5 Faculty of Theology, University of Ljubljana, Ljubljana, Slovenia; 6 Department of Radiation Oncology, Institute of Oncology Ljubljana, Ljubljana, Slovenia; 7 Department of Pediatric Endocrinology, Diabetes and Metabolic Diseases, University Children's Hospital, UMC Ljubljana, Ljubljana, Slovenia; 8 Rho Sigma Research & Statistics, Ljubljana, Slovenia; 9 Faculty of Criminal Justice and Security University of Maribor, Slovenia; 10 Faculty of Health Sciences, University of Ljubljana, Ljubljana, Slovenia; 11 University Hospital Centre Zagreb, Zagreb, Croatia; 12 Clinical Hospital Center Rijeka, Rijeka, Croatia; 13 Clinical Medical Centre Osijek, Osijek, Croatia; 14 Sisters of Charity Clinical Hospital Centre Zagreb, Croatia; 15 University Hospital Split, Split, Croatia; 16 Andrija Štampar School of Public Health, School of Medicine, University of Zagreb, Zagreb, Croatia

## Abstract

**Aim:**

To assess the differences in the way how Slovenian and Croatian health care professionals (HCPs) confront ethical dilemmas and perceive the role of hospital ethics committees (HECs).

**Methods:**

This cross-sectional, survey-based study involved HCPs from three Slovenian and five Croatian university medical centers (UMC). The final sample sizes were 308 (244 or 79.2% women) for Slovenia and 485 (398 or 82.1% women) for Croatia.

**Results:**

Compared with Croatian physicians, Slovenian physicians reported a higher share of ethical dilemmas regarding waiting periods for diagnostics or treatment, suboptimal working conditions due to interpersonal relationships in the ward, and end-of-life treatment withdrawal, and a lower share regarding access to palliative care and patient information protection. Compared with Croatian nurses, Slovenian nurses reported a lower share of ethical dilemmas regarding the distribution of limited resources, recognizing the patient’s best interests, and access to palliative care. Compared with Croatian other HCPs, Slovenian other HCPs reported a lower burden of ethical dilemmas regarding waiting periods for diagnostics or treatment, distribution of limited resources, and access to palliative care. When encountering an ethical dilemma, all HCPs in both countries would first consult their colleagues. Slovenian and Croatian HCPs recognized the importance of the HECs to a similar extent, but viewed their role differently.

**Conclusion:**

Croatian and Slovenian HCPs are confronted with different ethical dilemmas and perceive the role of HECs differently.

An ethical dilemma arises when we are confronted with a situation with two morally justifiable solutions, none of which is entirely satisfactory (1). In the course of their daily work, health care professionals (HCPs) encounter a broad range of ethical dilemmas (2-4), which often result in a moral distress for HCPs (5,6). A critical requirement for a successful response to an ethical dilemma is a strong foundation in medical professionalism cultivated during medical training and consolidated during professional work experience and career development (7-9).

Slovenia and Croatia, previously the westernmost republics of the former Yugoslavia and now European Union members, share the same historical, geopolitical, economic, and religious background. A recent survey in the largest Slovenian tertiary hospital, the University Medical Center Ljubljana, found that the most important contexts that give rise to ethical dilemmas among HCPs were waiting periods for diagnostics and treatment, suboptimal working conditions due to poor interpersonal relationships, and preserving patients' dignity, while the least important contexts were biomedical research, organ transplantation, and vaccine hesitancy (10). A study at the University Medical Center Rijeka found similar main ethical dilemmas in Croatian nurses and physicians, which included limiting life-sustaining therapy, euthanasia, and physician-assisted suicide (11).

Except these two studies, little to nothing is known about the ethical dilemmas of HCPs in Slovenia and Croatia. In response to this limited evidence, we conducted a prospective survey with a primary objective to assess the differences in the share of ethical dilemmas among different categories of HCPs (physicians, nurses, and other HCPs) in Slovenian and Croatian tertiary hospitals (university medical centers, UMCs). The UMCs were purposively selected because in this kind of hospitals, one encounters complicated cases usually referred from other health care institutions for complex diagnostic and therapeutic procedures, which can often raise ethical issues. The secondary objectives of our survey were to study differences in the opinion on the existence of standard procedures when HCPs are facing an ethical dilemma; to determine whom HCPs consult when facing an ethical dilemma; and to identify the opinion on the importance of hospital ethics committees (HECs) and their role in Slovenia and Croatia.

## Methods

### Study design

This cross-sectional, survey-based study involved Slovenian and Croatian HCPs in three Slovenian tertiary-level hospitals (University Medical Centers Ljubljana and Maribor, and University Hospital for Lung Diseases Golnik) and five Croatian tertiary-level University Medical Centers (University Medical Centers: Zagreb, Sestre Milosrdnice Zagreb, Osijek, Split, and Rijeka). We designed a questionnaire (see Supplement 1(Supplementary material)), translated it from Slovenian to Croatian, and backtranslated it to ensure semantic consistency in the understanding of the questions. We followed the conventional recommendations for trans-cultural translation and adaptation (12-14). The research was approved by the Slovenian National Ethics Committee and the Ethics Committee of each Croatian University Medical Center.

### Data collection

The hospitals were included in the study consecutively. The data were first collected at the UMC Ljubljana, from April to July 2015. The data from the other UMCs in Slovenia were collected in the autumn and winter of 2015, and in Croatia in the spring of 2016. We used a simple random sampling method for selecting the employees who met the inclusion criteria. The inclusion criterion was that participants were HCPs (physicians, nurses) and other HCPs, (laboratory technicians and engineers, radiological engineers, clinical psychologists, nurse assistants, biochemical technicians and engineers, pharmacists, social workers, physiotherapists, respiratory therapists, speech therapists, hygiene technicians, and psychologists). A comprehensive list of employees was obtained from the Human Resources Department of each hospital. According to the decision No. 090-59/2009 of the Slovenian Information Commissioner dated July 13, 2009, public employees are not entitled to expect privacy with regard to their names. Therefore, the employees' personal information can be acquired from employees if they decide to participate in the survey. The list was arranged by employees’ surnames in the alphabetical order. We informed the head and the head nurse of the clinical department of the hospitals by telephone and later by e-mails about the research objectives. The questionnaires were delivered to the Human Resources Department of each hospital and/or Secretariat at all clinical departments and services of each hospital personally or by internal mail. Departmental secretaries or employees from the Human Resources Department were directed to distribute the questionnaires to the selected HCPs. The anonymous responses to the questionnaires were then collected and put in designated envelopes, which were collected after two weeks and moved into a larger box. In this way, we ensured a complete anonymity of the participants.

In some hospitals, only personal registration numbers and professional profiles of HCPs were disclosed. Therefore, after we chose eligible HCPs according to their personal registration number, the Human Resources Department in each of these hospitals distributed the questionnaire to HCPs by using their personal registration number.

According to previous research results, the expected share of HCPs facing ethical dilemmas (very) often is 60% (10). If we want to detect the effect with the accuracy of 5 percentage points at the significance level α = 0.05 and with 80% power, 770 HCPs (per country) should be included in the study. The expected non-response rate of 30% increases the sample size by additional 230 HCPs.

Proportional stratified sampling was used to select HCPs. The anonymized list of HCPs with unique IDs was sent from each hospital. The proportional number of HCPs to be included in the sample was calculated for each hospital. The sample of HCPs for each hospital was selected with simple random sampling (the number of seed selection units in the sample was 02031979), performed with R 3.1.3, via function sample and with random seed set to the date of the received list of HCPs.

The response rates in Ljubljana, Maribor, and Golnik hospital were 55%, 44%, and 62% respectively. The final sample size was 308. The response rates in Zagreb, Sestre Milosrdnice Zagreb, Osijek, Rijeka, and Split were 36%, 59%, 46%, 36%, and 44%, respectively. The final sample size was 485.

### The questionnaire

The study-specific questionnaire (Supplement 1(Supplementary material)) consisted of 20 questions divided into three parts. The first part comprised eight demographic questions (on age, gender, profession, workplace, and work experience). The second part was aimed to determine how frequently HCPs were confronted with ethical dilemmas and how they rated interpersonal relations in the workplace. We were interested in how they solved the recognized ethical dilemmas and what they thought were the most important areas of responsibility of the HECs. The third part consisted of questions about the HEC in their institution. We aimed to determine the percentage of HCPs who were aware of the existence of the HEC and assess their opinion on how well it was integrated into everyday clinical practice.

Four out of 12 questions in the second and third part were five-point Likert-type questions with frequency labels. Four questions were yes/do not know/no questions. The remaining four were multiple choice questions with an optional open response. The questionnaire was anonymous. The completion took about 10 minutes. The questionnaire was accompanied by a written explanation of the study's background and purpose.

### Questionnaire validation and testing

The questionnaire was pretested on 35 HCPs at the UMC Ljubljana. Wu used the pretest results to adjust the sample size necessary for the measurement of the primary endpoint with a predetermined precision. We also removed the questions that were not answered during pretesting, ie, those that showed a lack of measurement sensitivity.

After translation to Croatian, ten experienced Croatian professors of health and social sciences read the questionnaire for understanding and consistency. The questions were corrected if understanding and consistency with the Slovenian questionnaire were not achieved.

### Statistical analysis

Means and standard deviations were calculated for numerical variables, and frequencies and percentages were calculated for categorical variables. The differences in the shares of HCP types in the sample and target population were evaluated by the χ^2^ test. The preliminary analysis showed no need for multilevel (mixed-effect) analysis as intercepts did not significantly vary between hospitals no matter which dependent variable was included in the regression model. The share of HCPs facing ethical dilemmas (very) often for Slovenia and Croatia and bootstrapped 95% confidence interval were calculated. The association between country and binary outcome variables was assessed using logistic regression analysis. The significance level was set to α = 0.05. All statistical analyses were performed using SPSS, version 23.0 (IBM Corp; Armonk, NY, USA).

## Results

Sample characteristics by country are summarized in Table 1. The Slovenian sample included 244 (79.2%) female HCPs and the Croatian sample included 398 (82.1%) female HCPs. The mean age (SD) in Slovenia was 40.1 (10.5) years and in Croatia 42.3 (11.1) years. The Slovenian sample included 51 (16.6%) physicians, 198 (64.3%) nurses, and 59 (19.2%) other HCPs. The corresponding numbers in Croatia were 102 (21%), 248 (51.1%), and 135 (27.8%). The shares of HCP types in both Slovenian (*P* = 0.033) and Croatian sample (*P* < 0.001) significantly differed from the population shares. In the Slovenian sample, physicians and other HCPs were underrepresented, while nurses were overrepresented (8 more physicians, 15 more other HCPs, and 22 fewer nurses were expected in a sample of the given size). In the Croatian sample, physicians were underrepresented, while nurses and other HCP were overrepresented (33 more physicians, 19 fewer nurses, and 53 fewer other HCP were expected) (Table 1).

**Table 1:** Healthcare professionals' (HCPs) characteristics by country. Data are presented as number (%) if not otherwise indicated

	Slovenia (n = 308)	Croatia (n = 486)
**Gender**		n = 485
male	64 (20.8)	87 (17.9)
female	244 (79.2)	398 (82.1)
**Mean age (SD; n)**	40.1 (10.5; 304)	42.3 (11.1; 484)
**Mean years of service (SD; n)**	18.0 (11.1; 308)	20.3 (11.7; 485)
**Mean years of service** **in the current hospital (SD; n)**	15.8 (10.8; 308)	18.4 (12.1; 483)
**Religion**	n = 251	n = 470
religious	213 (84.9)	437 (93)
not religious	38 (15.1)	33 (7)
others	1 (0.3)	0 (0)
**HCP**		n = 485
physicians	51 (16.6)	102 (21)
nurses	198 (64.3)	248 (51.1)
others	59 (19.2)	135 (27.8)
**Hospital workplace**		n = 485
reception clinic	17 (5.5)	10 (2.1)
clinic	48 (15.6)	59 (12.2)
emergency department	15 (4.9)	18 (3.7)
hospital ward	101 (32.8)	144 (29.7)
intensive care unit	34 (11)	51 (10.5)
diagnostic department	21 (6.8)	76 (15.7)
operating theater	44 (14.3)	69 (14.2)
other	28 (9.1)	58 (12)

The share of HCPs frequently encountering an ethical dilemma in Slovenia was 67.4% (95% CI 62.2%-72.3%), and in Croatia it was 81.6% (95% CI 78.1%-85.1%). In both countries, approximately 90% of physicians most frequently encountered an ethical dilemma during their work. The share of nurses encountering an ethical dilemma (very) often in Slovenia was 64.1% and in Croatia it was 81.7%. Slovenian nurses had a lower odds for encountering an ethical dilemma than Croatian nurses (OR 0.4; 95% CI 0.3-0.6). The share of other HCPs frequently encountering an ethical dilemma was higher in Croatia (73.9%) than in Slovenia (58.6%) (*P* = 0.037).

**Figure 1:**
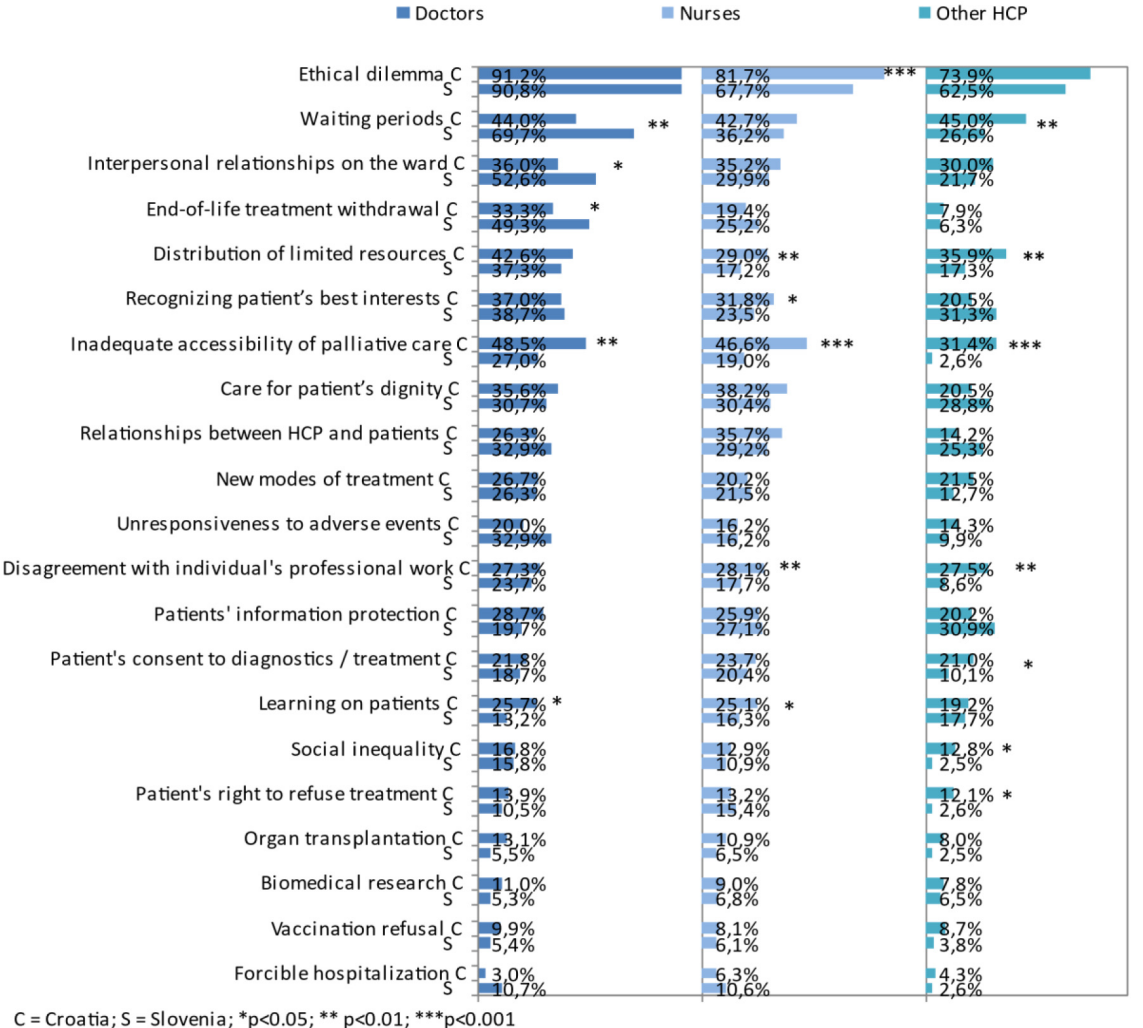
Frequent ethical dilemmas of healthcare professionals (HCPs) by country.

Different shares of Croatian and Slovenian physicians encountered ethical dilemmas regarding waiting periods for diagnostics or treatment, suboptimal working conditions related to interpersonal relationships in the ward, end-of-life treatment withdrawal, inadequate accessibility of palliative care, patient information protection, and forcible hospitalization (Figure 1). Long waiting periods for diagnostics or treatment posed an ethical dilemma for a higher share of Slovenian (68.6%) than of Croatian (44%) physicians. Slovenian physicians had 2.8 (95% CI 1.4-5.7) times higher odds of encountering this dilemma (Table 2). They also had a higher odds for encountering suboptimal working conditions related to interpersonal relationships in the ward (OR 2.2; 95% CI 1.1-4.3), end-of-life treatment withdrawal (OR 2.2; 95% CI 1.1-4.4), and forcible hospitalization (OR 5.3; 95% CI 1.3-21.5). The most frequent ethical dilemma for Croatian physicians was palliative care accessibility. Slovenian physicians had a lower odds for encountering this dilemma (OR 0.3; 95% CI 0.1-0.7). In comparison with Croatian physicians, Slovenian physicians had a lower odds for frequently encountering the dilemma of patient information protection (OR 0.3; 95% CI 0.1-0.9).

**Table 2:** The association between country and health care professionals' (HCPs) opinion on the existence of standard procedures when encountering ethical dilemmas (results of univariate logistic regression)

	Physicians				Nurses				Other HCPs			
	Croatia	Slovenia	odds ratio (95% confidence interval)	P	Croatia	Slovenia	odds ratio (95% confidence interval)	P	Croatia	Slovenia	odds ratio (95% confidence interval)	P
Yes	18 (18)	16 (32)	1 (0.4; 2.7)	0.988	45 (18.4)	71 (35.9)	1.5 (0.8; 2.8)	0.251	24 (18)	17 (28.8)	1.6 (0.4; 6)	0.493
No	17 (17)	15 (30)	Ref.		26 (10.6)	28 (14.1)	Ref.		9 (6.8)	4 (6.8)	Ref.	
Don't know	65 (65)	19 (38)	-	-	174 (71)	99 (50)	-	-	100 (75.2)	38 (64.4)	-	-

Slovenian nurses had a lower odds of encountering the ethical dilemmas of disagreement with individual professional work (OR 0.5; 95% CI 0.3-0.9), learning on patients (OR 0.5; 95% CI 0.3-0.8), distribution of limited resources (OR 0.4; 95% CI 0.3-0.7), recognizing patients’ best interests (OR 0.4; 95% CI: 0.3-0.7), care for the patient’s dignity (OR 0.5; 95% CI 0.3-0.8), relationships between HCPs and patients (OR 0.6; 95% CI 0.4-0.9), and accessibility of palliative care (OR 0.3; 95% CI 0.2-0.4). On the other hand, they had a higher odds for encountering forcible hospitalization (OR 2.2; 95% CI 1.1-4.4).

In comparison with Croatian other HCPs, Slovenian other HCPs had a lower odds for frequently encountering disagreement on individual’s professional work (OR 0.3; 95% CI 0.1-0.9), patient consent to diagnostics or treatment (OR 0.3; 95% CI 0.1-1), distribution of limited resources (OR 0.4; 95% CI 0.2-0.8), accessibility of palliative care (OR 0.1; 95% CI 0.02-0.4), and waiting periods for diagnostics or treatment (OR 0.4; 95% CI 0.2-0.9).

**Figure 2:**
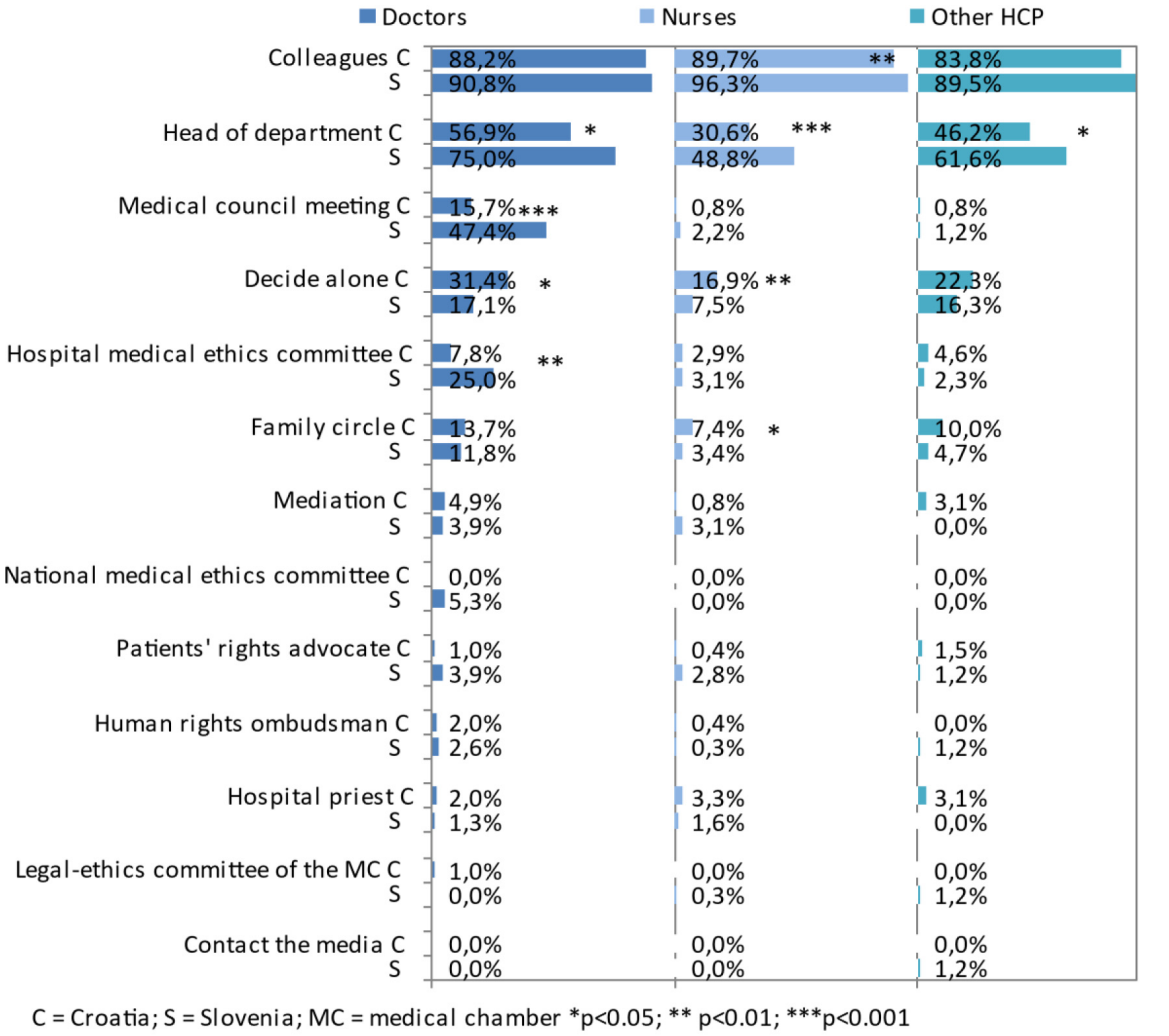
Healthcare professionals’ (HCPs) reaction when facing an ethical dilemma by country

When encountering an ethical dilemma, all HCPs from Slovenia and Croatia first consulted their colleagues (Figure 2). In comparison with Croatian physicians, Slovenian physicians had a higher odds for consulting the head of the department (OR 2.8; 95% CI 1.3-6), convening a medical meeting (OR 6.5; 95% CI 3-14.1), or discussing the dilemma with the hospital ethics committee (OR 6.4; 95% CI 2.5-16.1). On the other hand, they had a lower odds for deciding alone (OR 0.3; 95% CI 0.1-0.8).

Slovenian nurses had 3.1 (95% CI 1.3-7.4) times higher odds for consulting colleagues in comparison with Croatian nurses. They also had 1.6 (95% CI 1.1-2.4) times higher odds for consulting the head of department. On the other hand, they had a lower odds for discussing the ethical dilemma within their family circle (OR 0.4; 95% CI 0.2-1) or for deciding alone (OR 0.5; 95% CI 0.3-0.8). Slovenian other HCPs had a lower odds for deciding about the dilemma alone (OR 0.2; 95% CI 0.1-0.7).

Comparable shares of Slovenian and Croatian HCPs knew the answer to the question about the existence of standard procedures when encountering an ethical dilemma. In both countries, the share of HCPs who did not know the answer to the question was above 35% for physicians, above 50% for nurses, and above 60% for other HCPs. The share of “don’t know” answers was slightly higher in Croatia than in Slovenia for all three HCP groups (Table 2).

Physicians, nurses, and other HCPs in Slovenia and Croatia to a similar extent recognized the importance of the HEC (Table 3).

**Table 3:** The association between country and health care professionals' (HCPs) opinion on the importance of hospital ethics committee (results of univariate logistic regression)

	Physicians				Nurses				Other HCPs			
	Croatia	Slovenia	odds ratio (95% confidence interval)	P	Croatia	Slovenia	odds ratio (95% confidence interval)	P	Croatia	Slovenia	odds ratio (95% confidence interval)	P
Weaker agreement	31 (30.7)	12 (24)	Ref.		56 (22.9)	35 (17.8)	Ref.		26 (19.5)	8 (13.6)	Ref.	
Stronger agreement	70 (69.3)	38 (76)	1.4 (0.6; 3)	0.392	189 (77.1)	162 (82.2)	1.4 (0.9; 2.2)	0.189	107 (80.5)	51 (86.4)	1.5 (0.7; 3.7)	0.318

A higher share of Slovenian physicians than of their Croatian colleagues considered that the role of the HEC should be the provision of moral support for HCPs (OR 5; 95% CI 1.2-20.2). On the other hand, they ascribed lesser importance to other roles, such as developing protocols (OR 0.5; 95% CI 0.2-1) and improving communication (OR 0.4; 95% CI 0.2-0.9).

In comparison with Croatian nurses, a higher proportion of nurses in Slovenia believed the role of the HEC to be the revision of difficult cases (OR 1.8; 95% CI 1.2-2.7), resolving disagreements (OR 2.1; 95% CI 1.4-3.3), moral support for HCPs (OR 1.7; 95% CI 1-2.6), counseling hospital management (OR 3.6; 95% CI 1.5-8.2), and assessing new treatment modes (OR 4.5; 95% CI 1.8-11.5). They attached lower importance to staff education (OR 0.6; 95% CI 0.4-0.9) and improving communication (OR 0.4; 95% CI 0.3-0.6) as the roles of the HEC.

Other HCP from Slovenia in comparison with their Croatian colleagues attached higher importance to assessing new modes of treatment (OR 4.4; 95% CI 1.5-12.8) and lower importance to improving communication (OR 0.4; 95% CI 0.2-0.7) as the roles of the HEC.

## Discussion

This study, a continuation of the previous study by our research team among Slovenian HCPs at tertiary-level institutions (15), revealed several important findings. First, Slovenian and Croatian HCPs confronted ethical dilemmas (very) often (67.4% and 81.6%, respectively). However, while approximately 90% of physicians in both countries most frequently encountered ethical dilemmas, the share among nurses and other HCPs in Slovenia was lower (64.1% and 58.6%, respectively) compared with their Croatian colleagues (81.7% and 73.9%, respectively). Although Slovenia and Croatia have had similar postsocialist health care transitional problems before and after joining the European Union, Slovenian physicians compared with Croatian colleagues had higher odds for encountering ethical dilemmas related to long waiting periods for diagnostics or treatment, interpersonal relationships in the ward, end-of-life treatment withdrawal, and forcible hospitalization, and lower odds for encountering ethical dilemmas related to inadequate accessibility of palliative care and patient information protection. No differences were found in other 20 ethical dilemmas listed in the questionnaire. The lowest shares were observed for vaccination refusal, biomedical research, forcible hospitalization, and organ transplantation. Slovenian HCPs compared with Croatian HCPs had a 3.5 higher share of encountering the dilemma of forcible hospitalization (14.0% vs 3.0%).

The three most significant ethical problems were waiting periods for diagnostics and treatment, interpersonal relationship on the ward, and end-of-life treatment withdrawal. The first pertains to organizational and financial resources in terms of enough medical staff and health insurance payments available for very expensive diagnostics and treatments; the second concerns professionalism among HCPs and between them and the patients; while the third relates not only to ethical but also to decision-making processes in national legislation on patient’s rights and criminal law on treatment negligence. Overall, these data, despite differences between Slovenia and Croatia, are in concordance with other international studies, which showed that between 60% and 90% of HCPs encountered various ethical dilemmas during their work (10,11,15-18). Other researchers, however, did not ask two important questions – that about waiting periods for diagnostics and care of the patients and that about interpersonal relationships in the ward. The answers to these questions in our survey significantly differed between Slovenia and Croatia. Although both countries use a central, electronic ordering for diagnostics and hospital admissions, higher odds of encountering an ethical dilemma among Slovenian physicians might be attributed to shortage of facilities, human resources, and funds, despite government-funded medical expenses.

Similar shares of Slovenian and Croatian physicians confronted the distribution of limited resources, while this dilemma was encountered by significantly fewer Slovenian nurses and other HCPs compared with their Croatian counterparts. However, these two groups of HCPs did not encounter disturbed interpersonal relationships in the ward in a significantly different share compared with physicians. In contrast, Slovenian physicians encountered a much higher share of ethical dilemmas related to interpersonal relationships in the ward compared with Croatian physicians. Sorta-Bilajac et al (11) showed that the main ethical dilemmas among nurses and physicians were similar and related to limiting life-sustaining therapy, euthanasia, and physician-assisted suicide.

We cannot explain adequately why Croatian nurses and other HCPs reported higher shares of ethical dilemmas related to distribution of limited resources than Slovenian and Croatian physicians. We may only propose further research into the ethical dilemmas of nurses and other HCPs in both Slovenia and Croatia.

Palliative care accessibility was rated as a more important ethical dilemma among all Croatian HCPs compared with Slovenian ones. We can discuss this finding from two aspects if we know the organization and structure of palliative care in both countries (19,20). This observation is incongruent with a better organization of palliative care in Croatia, higher willingness of Croatian HCPs to provide palliative care, and their better opportunities for permanent education compared with the Slovenian situation (21,22). On the other hand, Croatia has much lower accessibility of beds for palliative care (23). Finding a definite answer would require a more in-depth survey on this subject in both countries.

In the past few years, Slovenian and Croatian nurses have been gaining the highest academic achievements. Accordingly, the nurses' professional relationship with physicians and other HCPs has dramatically changed. Nurses are not only involved in nursing care, but collaborate with physicians in many completely new jobs requiring special skills. They are in close contact with the patients, so it is not surprising that they are confronted with similar ethical dilemmas as physicians. Interpersonal relationships in the ward could be disturbed not only within the same group of HCPs but also between these groups, eg, between physicians and nurses or physicians and other HCPs (24-26). Our results show that a higher share of Croatian nurses and other HCPs encountered disagreement with individual’s professional work compared with their Slovenian counterparts; however this difference was not observed between Slovenian and Croatian physicians.

When facing an ethical dilemma, all Slovenian and Croatian HCPs reported that they would first consult their colleagues. Slovenian physicians had a higher odds of consulting the head of department, convening medical council meeting or discussing the dilemma with hospital medical ethics committee, compared with Croatian physicians. The same was true also for nurses and other HCPs. This indicates that the culture of convening a medical council meeting whenever HCPs are faced with an ethical dilemma is highly developed among Slovenian physicians. In our previous study, merely 60% of intensive care physicians reported knowing how to proceed when facing an ethical dilemma and 23% had consulted an HEC before. Furthermore, 42% of the respondents knew the name of the head of the HEC in their institution, whereas 17% of them reported not to have a HEC in their institution (27). At the same time, the share among pediatric intensivists was 91.7% (28), while the shares among residents and specialists in pediatrics were much lower (44.1%; 42.9%). More than half of the intensivists (54.2%) had sought advice from a medical ethics committee in the past, as compared with 12.0% and 12.1% of specialists and residents, respectively (28). These findings are in concordance with our results, which showed that 35.3% of Slovenian physicians consulted the HEC, compared with only 7.8% of Croatian physicians.

The role of HECs is well established (individual case consultations, education of HCPs, and policy formation) (29-31). Comparable shares of Slovenian and Croatian HCP knew the answer to the question about the existence of standard procedures when encountering an ethical dilemma. In both countries, however, the share of HCPs who did not know the answer to the question was above 35% for physicians, above 50% for nurses, and above 60% for other HCPs. The share of “don’t know” answers for all three HCP groups was somewhat higher in Croatia than in Slovenia.

Slovenian and Croatian HCPs to a similar extent recognized the importance of the HECs. Among all Slovenian HCPs, the lowest share of physicians believed that a role of HEC was improving communication. Contrary to our expectations, interpersonal relationships in the ward were a major ethical dilemma among Slovenian physicians, but not the Croatian ones. This could be explained by a low public opinion of Croatian physicians, who are often believed to put their own interests ahead of the patients’ (32,33). Nevertheless, a similar share of Slovenian and Croatian nurses and other HCPs believed that improving communication was a role of a HEC.

Our study is one of the first to involve different categories of HCPs employed in tertiary hospitals. This provided new insights into the variations in the ethical dilemmas experienced by HCPs in the two countries, including how HCPs respond to these experiences and how they rely on the HECs to resolve such dilemmas. At the same time, this study has a number of limitations. First, only HCPs with formal professional designations were included, while other hospital workers who perform supportive functions in the hospital operations were left out. Furthermore, the low response rates and underrepresentation of specific HCPs, notably physicians, may have resulted in selection bias. However, this possible bias could have been minimized due to similar low response rates and underrepresentation in both Slovenian and Croatian samples of physicians. Second, the study examined only the HCPs working in tertiary institutions, making the findings not generalizable to HCPs working in primary or secondary level institutions. Third, despite a strong validation process, as well as two previous studies that used the same questionnaire, some important ethical dilemmas could have been overlooked.

In conclusion, while Slovenia and Croatia share the same historical, geopolitical, economic, and religious background, they are confronted with different ethical dilemmas and perceive the role of HECs differently. Therefore, further studies are needed to determine the reasons for these differences.
