# Supplementary material for: How health care professionals confront and solve ethical dilemmas – a tale of two countries: Slovenia and Croatia
Source: Croat Med J. 2021 Apr;62(2):120–9. doi: 10.3325/cmj.2021.62.120 (PMC8107988; doi:10.3325/cmj.2021.62.120)
Supplement: Supplementary material [file CroatMedJ_62_s007.pdf]

Supplementary material: Questionnaire for healthcare professionals

The Role of Medical Ethics Committees in Healthcare Institutions in Slovenia

A SURVEY ON HEALTHCARE PROFESSIONALS' ETHICAL DILEMMAS AND THE ROLE OF THE MEDICAL ETHICS COMMITTEES

Dear healthcare worker,

This is a questionnaire accompanying the study entitled *Healthcare Professionals' Ethical Dilemmas and the Role of the Medical Ethics Committees in Healthcare Institutions in Slovenia.*

Ethics is the science of morality. Medical ethics in particular is concerned with applying ethical and moral principles in day-to-day challenges of the healthcare professional. Ethical dilemmas arise when the healthcare professional is faced with a choice between two or more opposing moral dilemmas.

We are aware that you yourself might have already confronted such dilemmas. We are therefore interested in learning more about your ability to recognize and resolve these situations in an ethically appropriate manner. Especially, whether you know where to seek support, whether you are satisfied with the way ethical dilemmas are currently being addressed in your hospital, and what your expectations are with respect to how such a system should operate. Medical ethics committees are recognized world-wide as a well-established means for resolving ethical dilemmas. With this survey, we intend to explore their role in Slovenian healthcare institutions. The study was approved by the National Medical Ethics Committee of the Republic of Slovenia.

We hope to see an enthusiastic response from the healthcare community. You can greatly help us in this by filling out this questionnaire; your participation in the study is entirely anonymous. The results will be made publicly available.

We thank you in advance for taking part in the study.

The questionnaire is protected by copyright law. Copying is prohibited. Reuse is allowed only with prior written approval by the study authors.

1. GENERAL INFORMATION

1.1. GENDER

- ☐ male
- ☐ female

1.2. AGE

Please fill in: _______ years

1.3. RELIGIOUS/WORLD VIEWS

Religions are listed in descending order according to the number of adherents from the 2002 census.

- ☐ Catholic
- ☐ Muslim
- ☐ Orthodox
- ☐ Protestant
- ☐ atheist
- ☐ undeclared
- ☐ I decline to answer
- ☐ other (please specify): ______________________________

1.4. TYPE OF INSTITUTION

- ☐ secondary level institution
- ☐ tertiary level institution
- ☐ other (please specify): ______________________________

1.5. EMPLOYMENT

- ☐ healthcare assistant
- ☐ nurse
- ☐ nursing graduate
- ☐ physiotherapist
- ☐ work therapist
- ☐ lab manager
- ☐ radiologist
- ☐ clinical psychologist
- ☐ medical trainee:
  - please specify area of specialization: ______________________________
    ____

- other (please specify): ____
  ____

1.6. IN WHICH HOSPITAL UNIT DO YOU SPEND MOST OF YOUR WORK TIME?
Please choose only one answer.

- ☐ reception clinic
- ☐ clinic
- ☐ emergency wards
- ☐ hospital wards
- ☐ intensive care units
- ☐ diagnostic wards
- ☐ surgical wards
- ☐ elsewhere (please specify): _______________________________

1.7. HOW LONG HAVE YOU BEEN EMPLOYED AT YOUR CURRENT HEALTHCARE INSTITUTION?

Please fill in: _______ years

1.8. WHAT IS YOUR TOTAL EMPLOYMENT PERIOD?

Please fill in: _______ years

2. MANAGING AND RESOLVING ETHICAL DILEMMAS

This section includes questions pertaining to ethical dilemmas in your day-to-day work and interpersonal relations in the workplace. Ethical dilemmas are listed in part 2.2.

2.1. HOW OFTEN ARE YOU FACED WITH ETHICAL DILEMMAS?

- ☐ very rarely
- ☐ rarely
- ☐ not frequently, not rarely
- ☐ frequently
- ☐ very frequently

2.2. PLEASE ESTIMATE HOW OFTEN YOU ENCOUNTER ETHICAL DILEMMAS IN THE DOMAINS LISTED BELOW IN YOUR PROFESSIONAL WORK. *Please indicate by putting a cross in the appropriate box.*

	very rarely	rarely	not frequently, not rarely	frequently	Very frequently
new modes of treatment and diagnostic procedures					

organ transplantation					
end-of-life treatment discontinuation					
suboptimal working conditions due to poor interpersonal relations at the department					
disagreement with an individual’s professional work					
lack of response to adverse events in patient management					
learning on patients					
social inequality or withdrawal of patient rights from base healthcare insurance					
biomedical research					
patient’s consent to participate in a diagnostic or therapeutic treatment					
allocation of limited resources					
recognizing patient’s best interest					
preserving patient’s dignity					
relations between healthcare professionals and the patient (or his/her relatives)					
forcible hospitalization					
patient’s right to refuse treatment					
refusal of vaccines					
insufficient availability of palliative care					
waiting times for diagnostic of therapeutic treatment					
other (please specify):					

2.3. WHAT DO YOU PROCEED WHEN FACED WITH AN ETHICAL DILEMMA? *It is possible to choose more than one answer.*

☐ I discuss it with the head of the department.
☐ I discuss it with co-workers.
☐ I call a medical council meeting.
☐ I consult the local medical ethics committee.
☐ I consult the national medical ethics committee (the National Medical Ethics Committee of the Republic of Slovenia)
☐ I consult the Committee for Legal and Ethical Issues of the Medical Chamber of Slovenia.
☐ I consult the patient’s legal representative.
☐ I consult the ombudsman.
☐ I consult the hospital chaplain.
☐ I try to resolve the issue with mediation.
☐ I try to resolve the issue through public discussion.
☐ I try to resolve the issue in family circles.
☐ I decide on my own.
☐ Other (please specify): ________________________________

2.4. DOES YOUR HOSPITAL MAINTAIN A STANDARD PROCEDURE FOR RESOLVING ETHICAL DILEMMAS?

- yes
- I don’t know
- no

2.5. CAN YOU RECALL A CASE FROM YOUR MEDICAL PRACTICE WHICH WOULD REQUIRE ETHICAL JUDGMENT BUT IT HAS IN FACT NOT RECEIVED IT?

- yes
- I don’t know
- no

2.6. PLEASE INDICATE WHAT MODE OF INSTRUCTION ON ETHICS AND MORALITY IN HEALTHCARE IS IN YOUR OPINION MOST IMPORTANT?

- self-study
- learning from more experienced peers
- family
- educational system or university
- workshops
- hands-on approach, learning through practice
- other (please specify): ______________________________

2.7. TO WHAT EXTENT DO YOU AGREE WITH THE FOLLOWING STATEMENT? *“The hospital staff needs regular formal trainings in healthcare ethics.”*

- I strongly disagree
- I disagree
- I neither agree neither disagree
- I agree
- I strongly agree

3. MEDICAL ETHICS COMMITTEE IN YOUR HEALTHCARE INSTITUTION

3.1. IN YOUR OPINION, HOW IMPORTANT IS THE ROLE OF THE MEDICAL ETHICS COMMITTEE IN RESOLVING ETHICAL DILEMMAS?

- not important at all
- not important
- neither important neither unimportant
- important
- very important

3.2. PLEASE CHOOSE FROM THE LIST BELOW THREE MOST IMPORTANT AREAS OF RESPONSIBILITY OF THE MEDICAL ETHICS COMMITTEE.

- review of difficult cases
- ethical consultation for employees (in session format)
- ethical consultation in the ward (with patients)
- education of healthcare staff
- improving communication
- conflict resolution
- preparation of guidelines or protocols
- support of patients in giving them a stronger voice in decision-making
- legal protection of physicians in decision-making process
- moral support for healthcare professionals
- allocation of limited resources
- counseling hospital management staff
- improving the quality of healthcare
- assessment upon introduction of novel treatment methods
- other (please specify): ______________________________

3.3. DOES THE HEALTHCARE INSTITUTION YOU ARE EMPLOYED WITH HAVE ITS OWN MEDICAL ETHICS COMMITTEE?

- yes
- I don’t know
- no

3.4. DOES THE HEALTHCARE INSTITUTION YOU ARE EMPLOYED WITH OFFER YOU THE POSSIBILITY TO HAVE ETHICAL CONSULTATIONS WITH THE MEDICAL ETHICS COMMITTEE?

- yes
- I don’t know
- no

3.5. HOW MANY TIMES DID YOU CONSULT THE MEDICAL ETHICS COMMITTEE OF THE HEALTHCARE INSTITUTION YOU ARE EMPLOYED WITH IN 2014?

Please fill in: _______ times
